# Visualization of Polymer Crystallization by In Situ Combination of Atomic Force Microscopy and Fast Scanning Calorimetry

**DOI:** 10.3390/polym11050890

**Published:** 2019-05-15

**Authors:** Rui Zhang, Evgeny Zhuravlev, René Androsch, Christoph Schick

**Affiliations:** 1 Institute of Physics and Competence Centre CALOR, University of Rostock, 18051 Rostock, Germany; rui.zhang@uni-rostock.de (R.Z.); christoph.schick@uni-rostock.de (C.S.); 2 Department of Polymer Science and Engineering, School of Chemistry and Chemical Engineering, Key Laboratory of High-Performance Polymer Materials and Technology of Ministry of Education, and The State Key Laboratory of Coordination Chemistry, Nanjing University, Nanjing 210093, China; 3 Shenyang Research Institute, Nanjing University, Shenyang 224300, China; 4 Interdisciplinary Center for Transfer-oriented Research in Natural Sciences (IWE TFN), Martin Luther University Halle-Wittenberg, 06099 Halle/Saale, Germany; rene.androsch@iw.uni-halle.de; 5 Butlerov Institute of Chemistry, Kazan Federal University, 18 Kremlyovskaya Street, Kazan 420008, Russia

**Keywords:** Fast Scanning Calorimetry (FSC), Atomic Force Microscopy (AFM), polymer morphology, crystal nucleation and growth

## Abstract

A chip-based fast scanning calorimeter (FSC) is used as a fast hot-stage in an atomic force microscope (AFM). This way, the morphology of materials with a resolution from micrometers to nanometers after fast thermal treatments becomes accessible. An FSC can treat the sample isothermally or at heating and cooling rates up to 1 MK/s. The short response time of the FSC in the order of milliseconds enables rapid changes from scanning to isothermal modes and vice versa. Additionally, FSC provides crystallization/melting curves of the sample just imaged by AFM. We describe a combined AFM-FSC device, where the AFM sample holder is replaced by the FSC chip-sensor. The sample can be repeatedly annealed at pre-defined temperatures and times and the AFM images can be taken from exactly the same spot of the sample. The AFM-FSC combination is used for the investigation of crystallization of polyamide 66 (PA 66), poly(ether ether ketone) (PEEK), poly(butylene terephthalate) (PBT) and poly(ε-caprolactone) (PCL).

## 1. Introduction

With the opportunity to cool and heat samples at extremely high rates, fast scanning chip calorimetry (FSC) has become an invaluable tool in materials research. It is now possible to gain information about the kinetics of the fast crystallization processes, crystal reorganization and melting, or the glass transition, as has already been summarized in several reviews [1,2,3,4,5,6,7]. Valuable information on crystal growth and even crystal nucleation can be gained. In particular, it has been shown that heterogeneous nucleation prevails on crystallization at low supercooling of the melt, often leading to spherulitic growth of lamellar crystals in polymers. By contrast, at high supercooling of the melt, homogeneous crystal nucleation occurs, which is often connected with a tremendous increase of the nucleation density by several orders of magnitude [2,8]. The increase of the nuclei density has implications for the crystalline-amorphous superstructure and causes, in some cases, dramatic changes of e.g., mechanical or optical properties, [9,10] justifying the intense research performed to understand the early stages of structure formation and the ultimate semi-crystalline morphology. In studies of the kinetics of nucleation by calorimetry, the change of the nuclei number during a thermal treatment is monitored via the change of the overall crystallization rate. Conversely, microscopic techniques offer unique opportunities to gain information about the number of nuclei by counting the formed crystals.

Optical microscopy, particularly with polarized light (POM), is one of the techniques that has been applied in crystallography for decades [11]. In combination, ex situ or in situ, with fast scanning calorimetry it provides deep insights into crystal nucleation and growth even for fast-crystallizing materials [1,2,5,12]. Nevertheless, the size of the crystals or crystal superstructures such as spherulites must be in the micrometer range to be visible. Electron microscopy, due to the much higher resolution, provides an alternative to optical microscopy [13,14]. Nowadays, high-resolution transmission electron microscopy (HRTEM) allows imaging with atomic resolution. Often, focused ion beam (FIB) is employed for HRTEM sample preparation [15]. Structures generated by fast controlled cooling in FSC become accessible in this way [16,17]. The drawbacks of HRTEM, conversely, are the enormous efforts required for sample preparation and possible sample damage by the electron beam [18], preventing its application to kinetic studies of organic materials. Nevertheless, Grapes et al., report an in situ combination of a dynamic TEM and nanocalorimetry [19]. Electron diffraction patterns were collected for a thin film during FSC experiments monitoring the formation of Al_3_Ni at heating rates up to 100,000 K/s [20]. For time-resolved scanning electron microscopy (SEM) less demanding sample preparation is required [19]. A combination of FSC with SEM was reported by LaVan et al., for the in situ observation of phase transitions even in polymers [21].

Another microscopic technique with molecular single chain resolution for polymers [22] is atomic force microscopy (AFM) [23]. AFM is an imaging technique, usually not requiring sophisticated treatments of the sample surface. In combination with a hot stage, AFM can access polymer crystallization on the lamellar scale in situ [24,25,26]. The formation of spherulites [27,28], recrystallization [29] and mesophase formation [30] were also shown. Chan’s group [28,31,32] used an AFM hot-stage combination to successfully follow the process of polymer crystallization in real time, starting from the unstructured melt, following the formation of lamellae and finally the growth of spherulites. They also showed how homogeneous and heterogeneous nucleation influence the crystallization process. However, Chan et al., used a polymer solution to match the time scales of the crystallization rate, the temperature-control performance of the used hot stage and the speed of the AFM image acquisition. Later on, Hobbs et al., studied crystallization of polymers employing an AFM with a fast scanner [24,33,34,35]. Meanwhile, Hobbs’s group applied fast scanning AFM to illustrate the growth of lamellae through video-like imaging (as images can be captured within 2–4 s), and the growth of individual lamellae was observed in real time [24,27].

Differential scanning calorimetry (DSC) with precise temperature control has been used to treat samples for AFM studies [36]. The use of DSC for sample preparation has several advantages. DSC allows for a more flexible temperature control than a hot-stage, enabling complex multi-step time-temperature experiments. Calorimetric data recorded during and after the heat treatment contain information about the morphology of the sample. Information about the non-crystalline part may be available from the glass transition, and the enthalpy and temperature of melting provide knowledge about the crystal fraction and crystal perfection, respectively. From cold crystallization on heating, information about the presence of crystal nuclei is accessible too. However, the slow heating and cooling rates of DSC (<10 K/s) limit its application, and particularly fast quenching and crystallization at deep undercooling of the melt is often not possible. Recent developments of chip-based fast scanning calorimeters [3], allowing heating and cooling rates up to 1,000,000 K/s, are therefore creating interesting opportunities for a combination with AFM.

Androsch et al., employed AFM ex situ in combination with FSC for the investigation of polymer crystallization [8,30,37,38]. After the thermal treatment of the sample, the sensor was taken out of the calorimeter and the membrane irreparably removed from the chip. The thin membrane with the sample was mounted on the AFM sample holder and the images were collected. As an intermittent step towards an in situ combination of FSC and AFM, Van den Brande et al., applied FSC and AFM to the crystallization of materials for organic electronics [39]. In this research, similar to Androsch et al., [30], the sample was first treated by FSC to allow crystallization under particular conditions. Afterward, the whole sensor was transferred to the AFM for imaging. Contrary to the sensors used by Androsch et al., the AFM images could be taken without destroying the chip sensor. Consequently, the same sample could again be thermally treated and imaged to illustrate the formed crystals [37,39]. Finding the same spot of the sample for imaging is difficult and therefore the growth of individual objects could not be followed.

In this study we describe an in situ combination of AFM with FSC. The aim of this combination is the collection of images from exactly the same spot of the sample, either after different thermal treatments or directly during isothermal annealing of the sample. The chip calorimeter sensor is used as the sample holder for the AFM and it is connected to the FSC electronics allowing fast heating and cooling as well as isothermal treatments of the sample. In other words, we describe a fast AFM hot stage with millisecond time resolution and the ability to perform calorimetric measurements in the AFM.

## 2. Materials and Methods

### 2.1. Combined AFM-FSC Device

**Figure 1:**
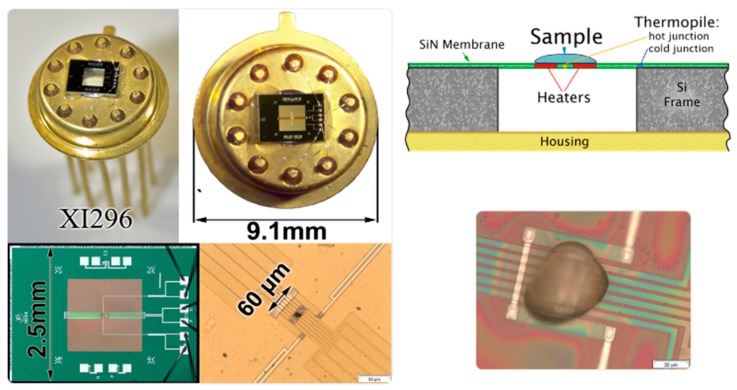
Schematic cross-section and zoomed-in view of the calorimeter chip XEN 39395 (XI296), Xensor Integration, Netherlands [45]. Reproduced with permission of [46].

**Figure 2:**
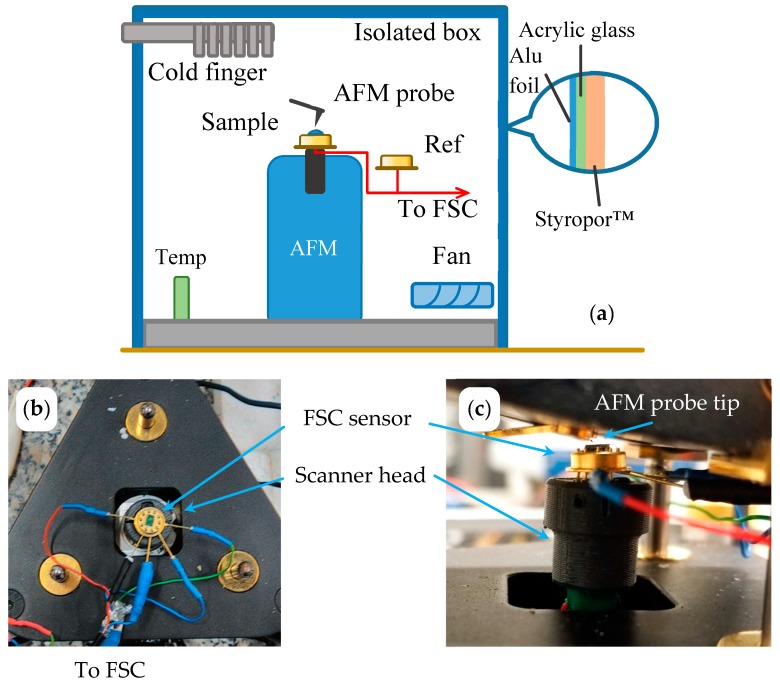
(**a**) Schematic view of the atomic force microscope fast scanning calorimeter (AFM-FSC) setup. (**b**) Photographs of the fast scanning calorimeter (FSC) sensor on the AFM scanner. (**c**) AFM probe approaching the sample on the sensor.

The AFM was a Level AFM from Anfatec, Oelsnitz, Germany. It was used in a non-contact mode with simultaneously recorded topology, phase angle and amplitude of oscillation [40]. We use standard silicon cantilevers NSC 14, Micromasch, Watsonville, CA, USA, with a resonance frequency of 110–220 kHz, tip radius of 8 nm, force-constant of 5 N/m and aluminum coating on the back side [41] or Scout 70 RAu, NuNano AFM probes, Bristol, UK, with a resonance frequency of 70 kHz, tip radius of 5 nm, force-constant of 2 N/m and gold coating on the back side [42]. The sample holder of the AFM on top of the scanner tube was replaced by a socket for the TO5 housing of the FSC chip. Chips from Xensor Integration, Delfgauw, The Netherlands, [43,44] mounted on a TO5 housing allowed for direct access of the sample by the AFM tip (Figure 1 and Figure 2).

A second empty calorimeter chip, the reference sensor, was placed next to the AFM inside a temperature-controlled box (Figure 2a). Both chip sensors were connected to the FSC electronics as described elsewhere [46,47].

For unperturbed operation of the FSC as well as the AFM, the open/uncovered sensor in the combined device needs to be decoupled from uncontrolled environmental influences such as temperature oscillations, air flow or sound waves. A protecting box made from 5 mm thick acryl glass sheets was placed over the AFM and thermally insulated with 30 mm foamed polystyrene (Figure 2a). The temperature inside the box was reduced to 278 K through a cold finger of a mechanical refrigerator, FT 100, Julabo, Seelbach, Germany. Nitrogen purge gas was used to ensure dry conditions.

The construction allowed mechanical stabilization of the chip sensor and the sample for high-quality AFM imaging. The amplitude signal was found to be less disturbed due to the droplet-shaped sample, therefore it was used in this work instead of the phase signal. A similar situation was described by Van den Brande et al., [39]. At the same time, though, FSC experiments can be performed without changing the position of the sample. The reduced temperature inside the protecting box slightly extended the range of materials for investigation and the cooling performance of the FSC. For interrupted crystallization experiments with collecting AFM images at the reduced ambient temperature, as shown in Figure 4b below, materials with a glass transition temperature (*T*_g_) near room temperature can be studied.

### 2.2. Sample Preparation and AFM Adjustment

**Figure 3:**
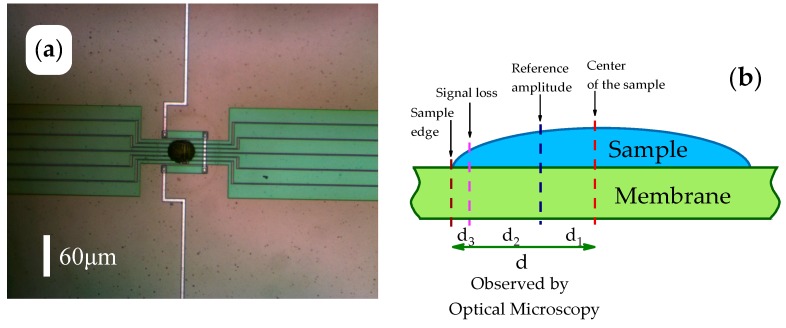
(**a**) Optical microscope image of a poly(ether ether ketone) (PEEK) sample on the chip-sensor. (**b**) Schematics of setting the reference amplitude of the cantilever for the AFM measurement.

Before mounting the chip sensor in the AFM, the sample was placed on the calorimeter chip sensor under an optical microscope as described elsewhere [48]. After mounting the sensor in the AFM, polymer samples were heated several times to above the melting temperature until a stable shape of the sample was reached. A first AFM image of the whole sample was then collected in non-contact mode at 278 K. Nearly flat surface areas near the center of the drop-like sample for more detailed studies were identified from this image, (see Figure 3).

The sample on the chip-sensor is generally close to a spherical cup, as shown in Figure 3a,b. The tip was placed next to the center of the sample, which is available from the optical micrograph, Figure 3a. Due to the limit of 2 µm for the Z-scanner (with Z being the vertical direction), the whole image of the sample with a height of about 10 µm was not available. Near the sample edge, the scanner lost the signal and imaging was not possible in the area labeled *d*_3_ in Figure 3b. In order to increase the accessible area, the reference amplitude was set at a place outside the center at *d*_1_. The corresponding AFM images and further detail on AFM adjustment can be found in Supplementary Materials A1–A3.

### 2.3. Materials

The details of the studied polymer samples are listed in Table 1. Materials were used as received without further treatment, if not stated otherwise.

### 2.4. Measurement Strategy

**Figure 4:**
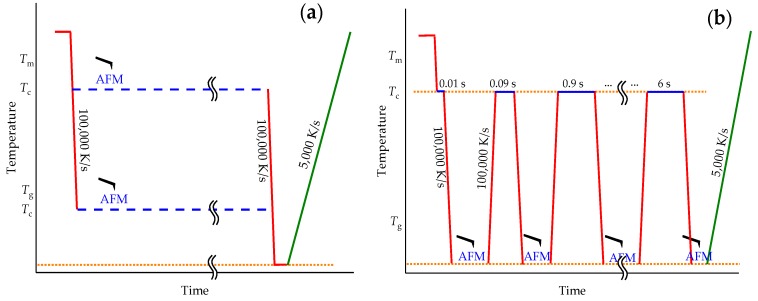
Program for studying isothermal crystallization by AFM-FSC combination. (**a**)—taking AFM images during slow crystallization at a temperature either close to $T_{m}^{0}$ or, using quenching, close to *T*_g_. (**b**)—time-resolved imaging, using ultra-fast cooling and heating ability of FSC. In both cases, the final FSC heating scan after crystallization is taken at 5000 K/s starting at ambient temperature.

The AFM is relatively slow, compared to FSC. The AFM used in this work needs about 30 min to collect a single image with the needed resolution of 512 × 512 pixel per image. True in situ imaging is therefore limited to slow processes such as crystallization near the equilibrium melting temperature ($T_{m}^{0}$) or near the glass transition (*T*_g_). In the latter case, combination with the fast cooling capabilities of the FSC are advantageous since the maximum of the crystallization rate can easily be bypassed for most polymers. A typical temperature–time profile for such studies is shown in Figure 4a. It consists of melting the sample by heating it to above $T_{m}^{0}$, quenching to the crystallization temperature (*T*_c_), annealing for several hours and collecting the AFM images, cooling and final heating. The cooling steps, if possible, are performed at rates far above the critical cooling rate to avoid any crystallization or nucleation [2]. The final heating scan, in contrast, is performed at a rate which gives the best results regarding detection of the temperature and enthalpy of melting for the used sensor and the sample under investigation.

Since crystallization must be slow to allow in situ AFM imaging, there is no useful calorimetric signal during the isotherm. The final heating scan is therefore used to determine the crystallinity from the enthalpy of fusion and the crystal stability from the melting temperature. If a rate of 100,000 K/s is not fast enough to prevent further crystallization on cooling below *T*_c_, as for polyethylene [53], heating can start directly from the annealing temperature. In this case melting may partially occur in the transient stage at the beginning of the FSC scan when the programmed heating rate is not yet reached.

If crystallization is fast and in situ observation is not possible, the fast cooling capability of the FSC allows for a different experiment approach, as shown in Figure 4b. As before, the sample is first heated to above $T_{m}^{0}$ and then quenched to *T*_c_. After an appropriate annealing time, crystallization is interrupted by cooling the sample at overcritical rate to a temperature significantly below the glass transition. If this temperature is low enough for the studied polymer, then crystallization is fully stopped and there is sufficient time to collect AFM images with high resolution. After taking the AFM image, the sample is quickly heated back to *T*_c_ and crystallization is allowed to proceed for a certain time. Then the sample is again quenched and the next image is taken. This procedure is repeated until the sample is fully crystallized or any intermediate stage of interest is reached. More complex temperature–time profiles are possible, as shown in Section 3.3 below. The final heating scan always allows for quantifying the reached crystallinity. The advantage of this method is that it provides enough time for AFM imaging after only 1 ms annealing, which is not accessible directly, not even with a video type AFM.

For quantification of crystallinity of the sample, its mass (*m*) was determined from the measured heat capacity step height at the glass transition temperature of the fully amorphous sample and the known step height in the specific heat capacity [54]:

(1) $$m=\frac{\Delta C_{p}}{\Delta c_{p}}=\frac{\Delta \Phi }{ß\Delta C_{p}}$$

where ΔΦ is the measured heat flow step at the glass transition of the fully amorphous sample, *ß* is the heating rate and Δ*c*_p_ is the step in specific heat capacity at the glass transition.

Crystallinity was determined as the ratio between the measured heat of fusion and the known heat of fusion of the 100% crystalline polymer:

(2) $$X=\frac{\Delta H}{\Delta H^{\infty }}$$

where Δ*H* is the measured heat of fusion and Δ*H*^∞^ is the heat of fusion of the fully crystalline material, taken from the ATHAS data bank [55].

## 3. Results

### 3.1. Crystallization at Low Undercooling

#### 3.1.1. In Situ Study of PCL Crystallization

As a first example, we present images in situ collected during expected slow crystallization of PCL at low undercooling. Even though the anticipated results were not obtained, we present this example to highlight specific observations of in situ AFM during crystallization and, next, show how fast scanning calorimetry in combination with AFM can overcome these limitations in an elegant and advantageous way.

**Figure 5:**
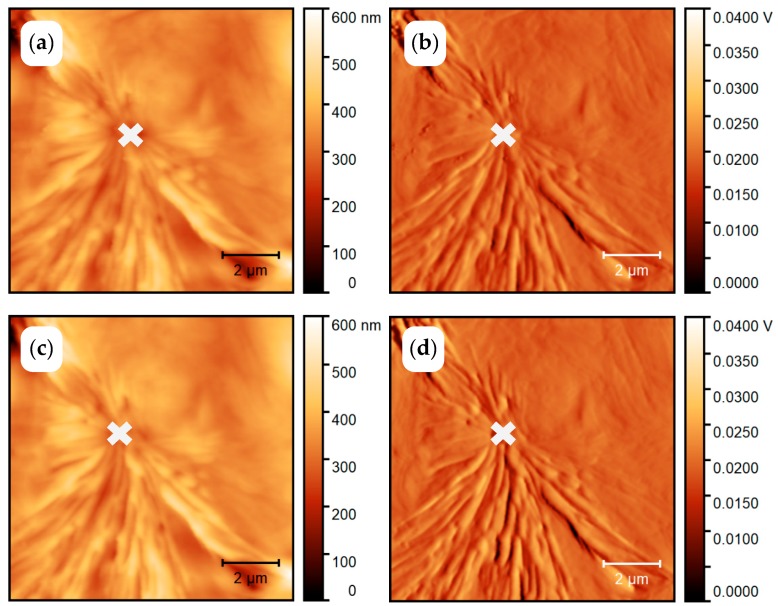
AFM images of poly(ε-caprolactone) (PCL) annealed at 325 K. The left column shows the topology and the right column shows the amplitude images. Images (**a**,**b**) are collected between 1000 and 3000 s and images (**c**,**d**) between 3000 and 5000 s after the quench.

The PCL sample was thermally treated, as shown in Figure 4a. It was first heated to above the melting temperature at 450 K and quenched to *T*_c_ = 325 K. According to Zhuravlev et al., [56] a primary crystallization half time of 10^5^ s is expected for crystallization at 325 K. With about 2000 s needed to capture the AFM image, it should be possible to collect about 50 AFM images before half of the sample is crystallized. Figure 5 shows the AFM images collected at 325 K between 1000 and 3000 s (Figure 5a,b) and between 3000 and 5000 s (Figure 5c,d) after the quench from the melt. Unexpectedly, the images are almost identical.

A spherulitic structure can already be seen in Figure 5a,b but no additional structures are formed during further annealing for half an hour (Figure 5c,d), suggesting that crystallization almost finished after 3000 s. However, this result differs significantly from the crystallization kinetics of PCL reported by Zhuravlev et al., [56]. Fortunately, the combined AFM-FSC device offers the possibility to identify the reason for this discrepancy. According to the temperature time profile of Figure 4a, the FSC heating scan has been recorded after taking the AFM images of Figure 5.

**Figure 6:**
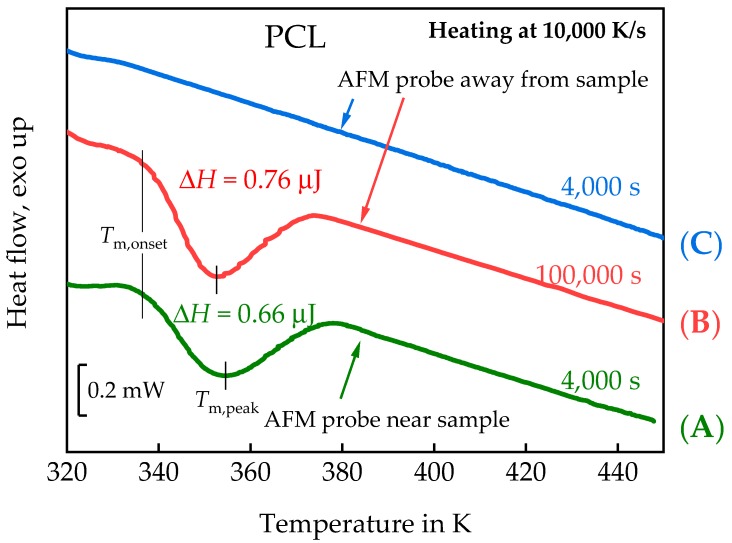
FSC heating scans of PCL after annealing at 325 K at different conditions regarding the placement of the AFM tip. Curve (**A**)—After collecting the images of Figure 5 (5000 s at 325 K, AFM in non-contact mode, about 5–10 nm distant to the sample). Curve (**C**)—same annealing time at 325 K as in case of curve (**A**) but with the AFM tip about 100 µm away from the sample. Curve (**B**)—AFM probe far away from the sample and annealing time at 325 K increased to 100,000 s.

In accordance with the AFM images, a significant crystallinity developed within the total time of 5000 s at 325 K. A melting peak of 0.66 µJ is seen in Curve (A) of Figure 6. Removing the AFM probe and annealing the PCL at 325 K for the same time does not result in a measurable melting peak, Curve (C) in Figure 6. A comparable melting peak of 0.76 µJ was only observed after annealing for 100,000 s at 325 K with the AFM probe far away from the sample surface, curve (B) in Figure 6. The roughly similar peak positions (*T*_m,onset_ ca. 337 K; *T*_m,peak_ ca. 353 K) indicate comparable lamellae thicknesses for the crystals grown at 325 K, independent of the nucleation pathway. In order to determine the sample mass, the sample was tested by FSC with a liquid nitrogen cooling system. The glass transition step of the fully amorphous sample yields the sample mass of ca. 14 ng. Crystallinity estimated by equation (2) were ca. 30% and 35% for curve (A) and curve (B), respectively. Here we used specific heat of fusion of fully crystalline material from the ATHAS databank: Δ*h*^∞^ = 156.82 J/g. Nucleation kinetics dominates the crystallization kinetics at the low undercooling of about 15 K in these experiments and the AFM probe near the surface obviously enhances nucleation.

The position of the nucleus of the growing spherulite is marked by a cross in Figure 5. This position is the place where the AFM tip initially approaches the sample surface before imaging starts in horizontal lines from the top of the images. The approach of the AFM tip to the surface takes about 1000 s. During this time a nucleus is formed, even if the AFM tip does not touch the sample surface. The reason for this localized nucleation is not yet known. At least two possible effects must be considered. On the one hand, the AFM tip locally cools the sample surface and may enhance formation of a nucleus just at the place of lowest temperature. On the other hand, the tip interacts through Van der Waals forces with the sample, which are of the order of 10^−12^ N for a distance of 5 to 10 nm between the AFM tip and the sample. AFM-tip-induced nucleation is a known phenomenon and discussed in several publications, e.g., [57].

Even if the interaction between AFM tip and sample is very weak, it is strong enough to influence the crystallization behavior of polymers, making an in situ investigation of isothermal polymer crystallization at the crystallization temperature difficult. Fortunately, the combination with the FSC allows for well-defined interruption of the crystallization process and collecting images at low temperatures where crystallization does not proceed, see Figure 4b. This strategy is next applied to follow spherulite growth in PEEK.

#### 3.1.2. Linear Growth Rate of PEEK Spherulites

**Figure 7:**
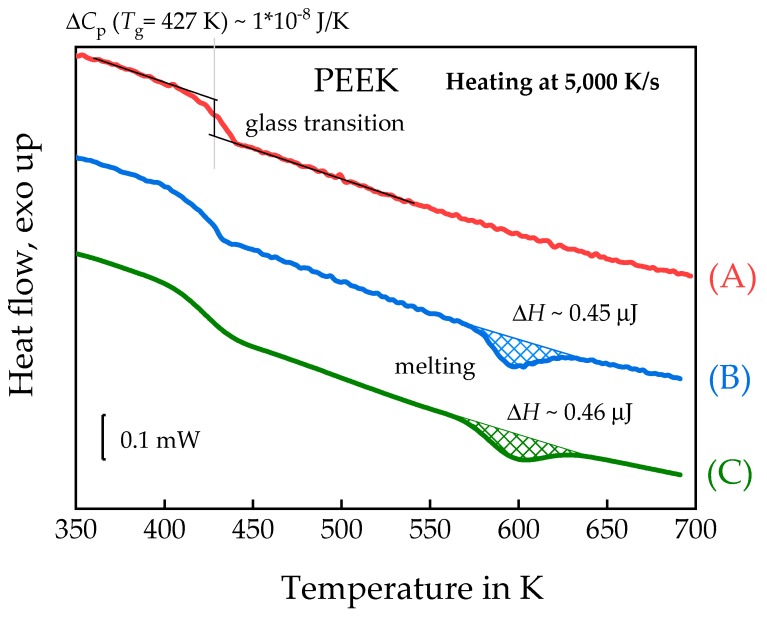
FSC heating curves of PEEK recorded using a rate of 5000 K/s. (**A**)—after quenching the melt to 300 K, (**B**)—after interrupted annealing at 564 K for a total time of 10 s and (**C**)—after annealing at 564 K for 10 s without interruptions, according to the temperature–time program in Figure 4b and Figure 4a, respectively.

Poly(ether ether ketone) (PEEK) is an intermediately fast crystallizing polymer but too fast to allow in-situ AFM imaging during crystallization at temperatures near the maximum of the crystallization rate at about 500 K. Nevertheless, the interrupted crystallization, as illustrated in Figure 4b, allows for a detailed investigation of spherulitic growth on length scales below that accessible by POM. The PEEK sample was cooled from 680 to 564 K at 100,000 K/s and then annealed for 0.01 s at this temperature. Then it was cooled down to 300 K at 100,000 K/s to take the AFM image. The 300 K is significantly below *T*_g_ of about 430 K, avoiding any crystallization during collection of the AFM image. After taking the AFM image, the sample was heated at 100,000 K/s back to 564 K and crystallization was allowed to continue for another 0.09 s. The next AFM image was again taken after quenching at 300 K. Consequently, the total annealing time before collection of the second image was 0.1 s. This way, AFM images of the PEEK sample were taken after cumulative annealing times of 0.01, 0.1, 1, 2, 4 and 10 s. Finally, after taking all AFM images, the sample was heated to 680 K and the melting curve was recorded, as shown in Figure 7 curve (B). Curve (A) of Figure 7 shows the heating scan after quenching the melt to 300 K, verifying the amorphous state of the sample. For this sample *T*_g_ was about 427 K. Sample mass was about 39 ng, determined from equation (2), using the measured heat capacity step at the glass transition Δ*C*_p_ (*T*_g_ = 427 K) = 10 nJ/K,. Crystallinity from curves (B) and (C) were ca. 9%. The values taken from the ATHAS databank were: Δ*h*^∞^ = 130 J/g and Δ*c*_p_ = 0.254 J/(g·K).

**Figure 8:**
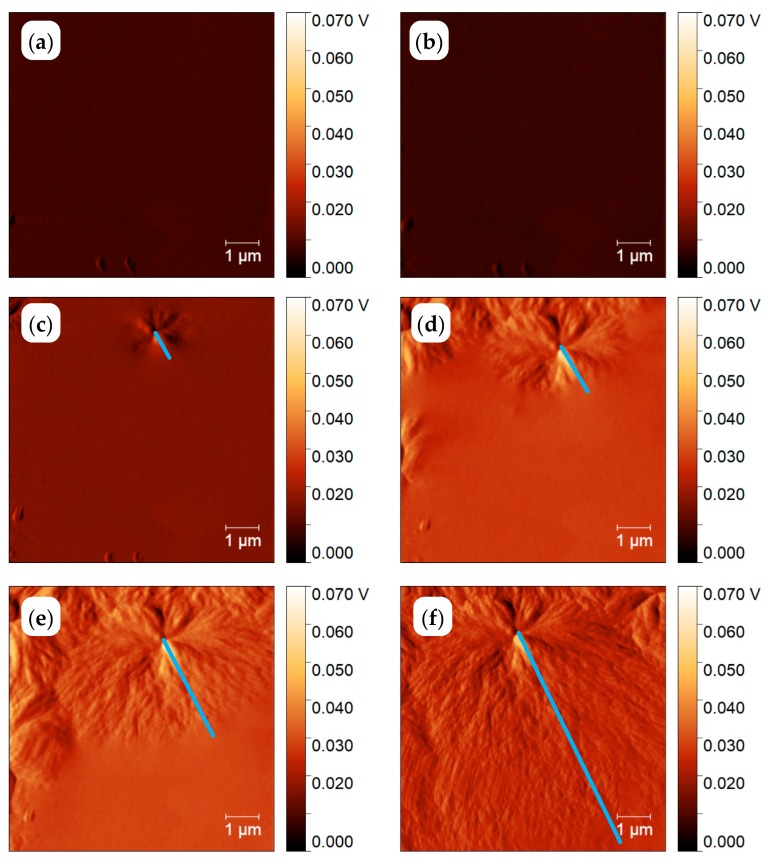
AFM amplitude images showing an area of 8 × 8 μm^2^. PEEK annealed at 564 K for 0.01 s (**a**), 0.1 s (**b**), 1 s (**c**), 2 s (**d**), 4 s (**e**) and 10 s (**f**). The reason for the slight asymmetry of the growth rate in direction of the blue line and perpendicular to it, images (**d**,**e**), is not yet known and was not investigated.

**Figure 9:**
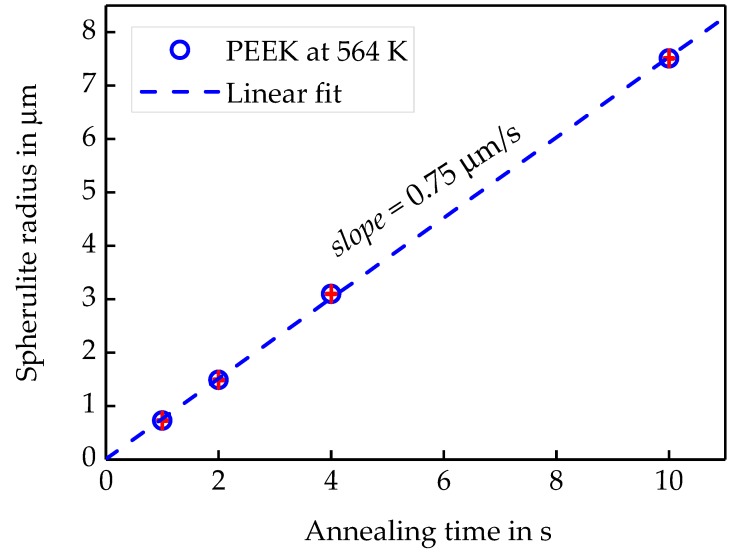
Surface spherulite radius vs. annealing time for PEEK at a sensor temperature of 564 K by AFM-FSC.

Figure 8 shows the AFM images, collected at 300 K, after annealing for the different annealing time at 564 K, as described above. After one second of annealing, the first spherulites show up. With increasing annealing time, the size of the spherulites is increasing, and the spherulites are nearly surface filling after 10 s annealing at 564 K. Since crystallinity from the curves in Figure 7 is below 10%, space is filled only at the surface but not in the bulk. The combined AFM-FSC device allows for following the growth of one and the same spherulite as illustrated with the images of Figure 8. Even the sample is quickly heated and cooled to and from the annealing temperature, the image always shows the same spot. The high reproducibility of the position of the AFM tip allows for measuring the size of the spherulites as a function of annealing time. The blue lines in Figure 8 correspond to the distance between the center and the edge of the investigated spherulite. This way the spherulite radius as a function of time was determined and the growth rate equals the slope of the line in Figure 9.

**Figure 10:**
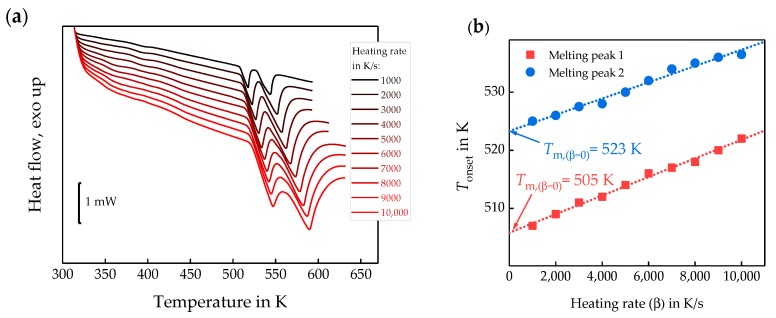
(**a**)—FSC heating scans of a tin particle placed on top of the PEEK sample, yielding the high temperature melting peak, and a tin particle placed beside the sample directly on the sensor membrane, yielding the low temperature melting peak. (**b**)—Melting peak onset temperatures against scanning rate. Tin sample on sensor (red squares) and tin sample on top of the PEEK sample (blue circles).

The slope of the data in Figure 9 yields a linear growth rate of 0.75 ± 0.01 µm/s at a sensor temperature of 564 K. The observed linear growth rate is much higher than expected from the POM data reported by Marand et al., [58]. There are several possible explanations for this: (i) Crystal growth is faster at the surface compared to the bulk due to enhanced surface mobility [59]. (ii) The surface of the sample on the FSC chip is colder than the membrane, where the temperature is measured [46]. To check if (ii) could explain the discrepancy, the surface temperature of the FSC sample was measured. A possible temperature gradient perpendicular to the sensor membrane is caused by a heat flow from the heated membrane to the cold gas in the surrounding [6,46,60]. To measure the actual temperature at the top of the sample, a tin particle has been placed on top of the sample, and the sample was heated and cooled at various rates spanning from 1000 to 10,000 K/s, Figure 10a. The extrapolated peak onset temperatures are plotted versus heating rate in Figure 10b.

**Figure 11:**
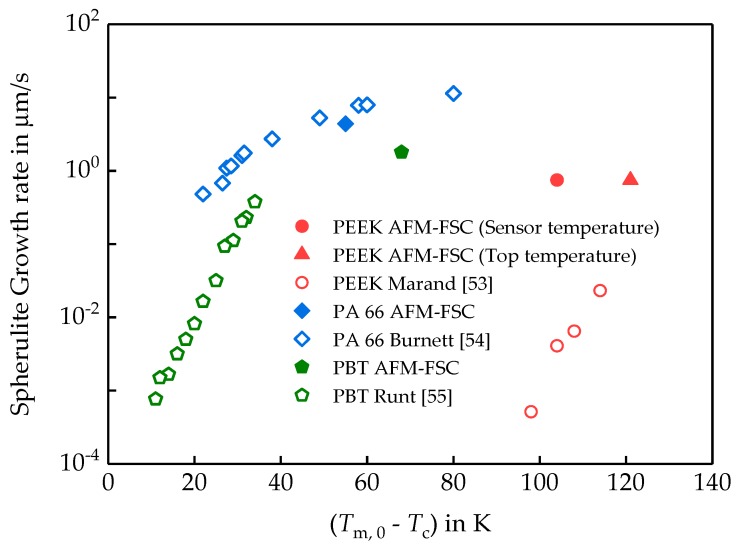
Spherulite growth rates of different polymers measured by AFM-FSC and polarized light (POM) as a function of undercooling below $T_{m}^{0}$. The corresponding AFM images are shown in Supplementary Materials A3. The equilibrium melting temperatures were taken from Table 1. Growth rates from POM were taken from the literature: PEEK [58], PA 66 [62], and PBT [63]. Throughout, filled symbols represent data obtained by AFM-FSC, while open symbols were taken from the literature, gained by POM.

The extrapolation to zero heating rate of the melting peak onset temperature [61] provides apparent melting temperatures of 505 and 523 K, corresponding to the tin sample on the membrane and on top of the PEEK sample, respectively. This indicates an 18 K temperature gradient between the sample top and bottom at tin melting temperature. In this case, since spherulites at the top of the sample are investigated, the crystallization temperature in this study is 546 K, when assuming a linear T-dependence of the offset. Considering the surface temperature as 546 K, the spherulite growth rate is in agreement with the POM data in Figure 11.

Compared to classical POM spherulite growth rate analyses, AFM allows for following spherulite growth at earlier stages, when the structure is too small for POM imaging. Furthermore, the FSC curves after a total annealing time of 10 s were also recorded and are shown in Figure 7. If required, heating scans can be recorded after shorter annealing times, but then the experiment must start from the very beginning if the final stage is of interest too.

For PBT and PA 66, the growth rates determined from the AFM images after crystallization at 450 and 480 K, respectively, partially agree with the POM data from the literature. The discrepancy may be explained by the difference in spherulite detection. AFM probes only the surface, while POM may see the whole spherulite. Another reason for the error can be the temperature gradient in the sample. Temperature gradient is especially large for high temperatures of crystallization e.g., for PEEK. The PEEK, crystallizing at 564 K sensor temperature and the growth rate gained from AFM considering the sensor temperature is significantly higher if compared to the POM data. But, considering the top surface temperature as described above, the AFM data follow the expected trend.

### 3.2. Semi-Crystalline Morphology of PA 66 at High and Low Supercooling

To further test the performance of the combined AFM-FSC device, we repeated an experiment reported by Gohn et al., [37]. Polyamide 66 (PA 66) was crystallized for 1000 s at 350 and 480 K, respectively. The experiment follows the scheme of Figure 4a, however, with taking the image at ambient temperature as in Figure 4b. The isotherm was chosen long enough to allow for the completion of crystallization. The sample was cooled at 100,000 K/s from 580 to 480 or 350 K, respectively, annealed for 1000 s and afterwards cooled to 278 K for AFM imaging. This temperature is below the *T*_g_ of PA 66 of about 323 K [64] and allows imaging without further crystallization. Finally, the sample was re-heated to 580 K to investigate the melting of the formed crystals.

**Figure 12:**
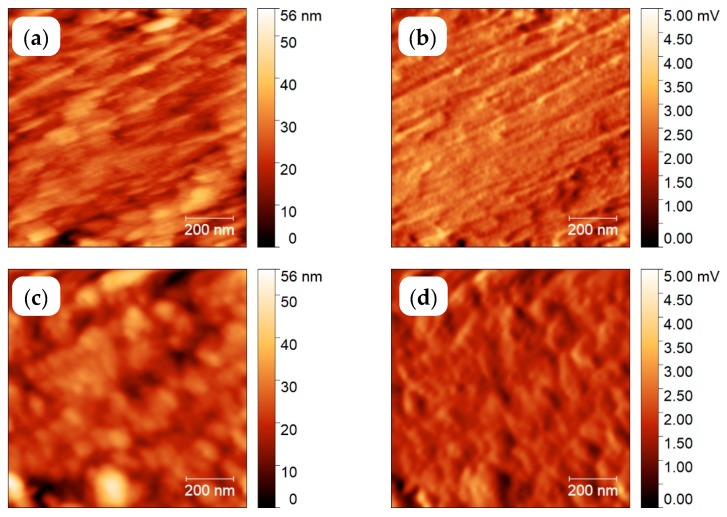
Topology (left) and amplitude (right) images taken at 278 K after annealing the melt of polyamide 66 (PA 66) for 1000 s at 480 K (**a**,**b**) and 350 K (**c**,**d**).

The AFM images of topology, left, and amplitude, right, of PA 66 crystallized at the two annealing temperatures are shown in Figure 12. After crystallization at 480 K, lamellae are observed; however, in the same scanning area of this sample, after crystallization at 350 K the lamellae are replaced by non-lamellar particle-like objects, with an apparent size of about 30–50 nm. If these objects are composed of smaller domains as discussed by Baer et al., [65] or the image is influenced by tip-smearing [66], it is outside the scope of the present study. Nevertheless, this result is similar to the result by Gohn et al., [37]. Unlike their study, which requires destruction of the sensor for collecting the AFM image, here, the two images have been taken from the same sample at the same area, only after different successive thermal treatments performed by FSC in the AFM.

**Figure 13:**
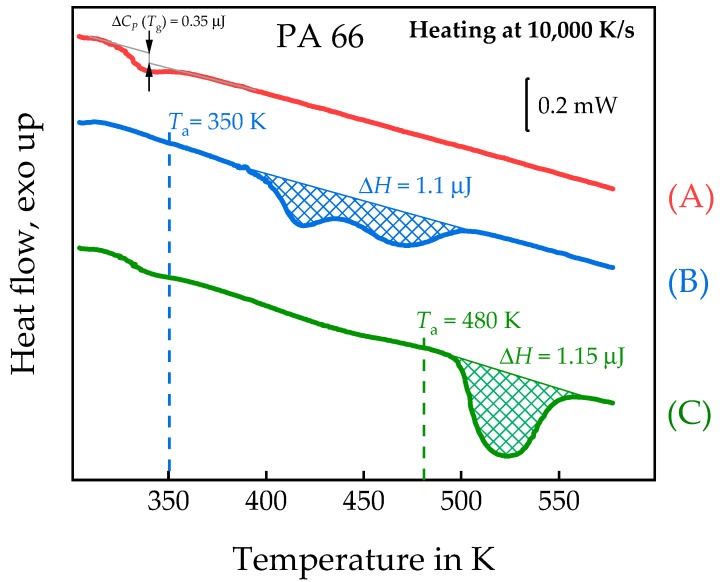
FSC melting curves of PA 66: (**A**)—quenched sample, (**B**)—annealed at 350 K for 1000 s, (**C**)—annealed at 480 K for 1000 s.

After taking the images shown in Figure 12, the samples were melted by heating to 580 K at 10,000 K/s, with the corresponding FSC curves shown in Figure 13. After annealing at 350 K a double- and after annealing at 480 K a single-melting peak is observed. The double melting peak after annealing at 350 K was not further evaluated, but may be explained as suggested by Furushima et al., in case of reorganization [67]. From equations 1 and 2, the sample mass was estimated to be ca. 7 ng and the crystallinities achieved at 350 K and 480 K ca. 62% and 64%, respectively. The values taken from the ATHAS databank were: Δ*h*^∞^ = 145 J/g and Δ*c*_p_ = 0.4 J/(g·K). Figure 13 shows the very different melting traces of the two samples, as expected from the differences in the AFM images of Figure 12.

### 3.3. Homogenous Crystal Nucleation in PBT

In order to perform a quantitative study of homogeneous crystal nucleation of PBT, a temperature profile resembling Tammann’s two stage nucleation and development scheme was applied [68].

**Figure 14:**
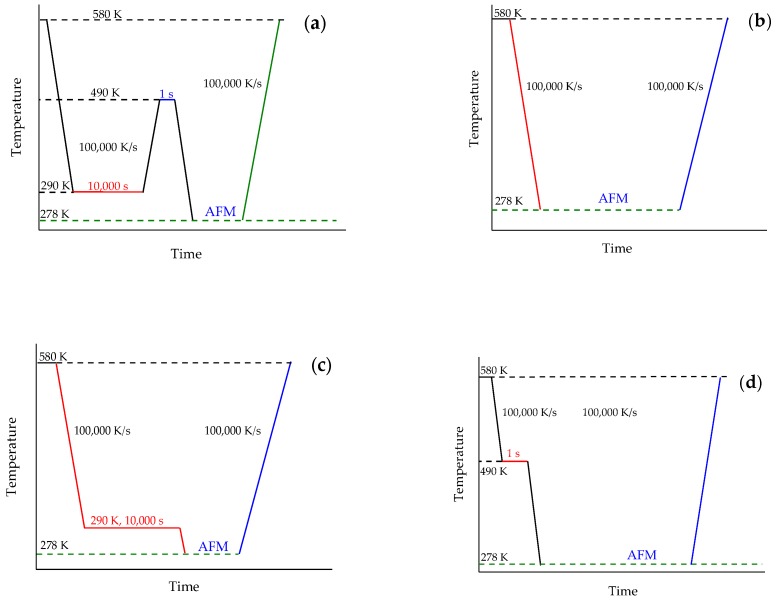
Temperature profile for nucleation studies of poly(butylene terephthalate) (PBT). All cooling and heating scans were performed at 100,000 K/s. (**a**)—Tammann’s two-stage nuclei development method. PBT was first cooled to the nucleation temperature of 290 K, and nucleated for different times. Then the sample was heated to the growth temperature of 490 K and crystal growth from existing nuclei was allowed for 1 s. The corresponding images were taken at ambient temperature. After each nucleation experiment, the sample was heated to 580 K for observation of the crystallinity by the melting enthalpy. (**b**)—quenching to ambient temperature of 278 K and imaging of the fully amorphous sample; (**c**)—quenching to the nucleation temperature of 290 K, annealing for 10,000 s and taking an image at 278 K; (**d**)—melt-crystallization at 490 K for 1 s, followed by imaging at ambient temperature.

Figure 14a shows the temperature–time program realizing Tammann’s two stage nucleation and development scheme in the AFM-FSC device. The other figures show temperature profiles for supporting experiments as explained below.

**Figure 15:**
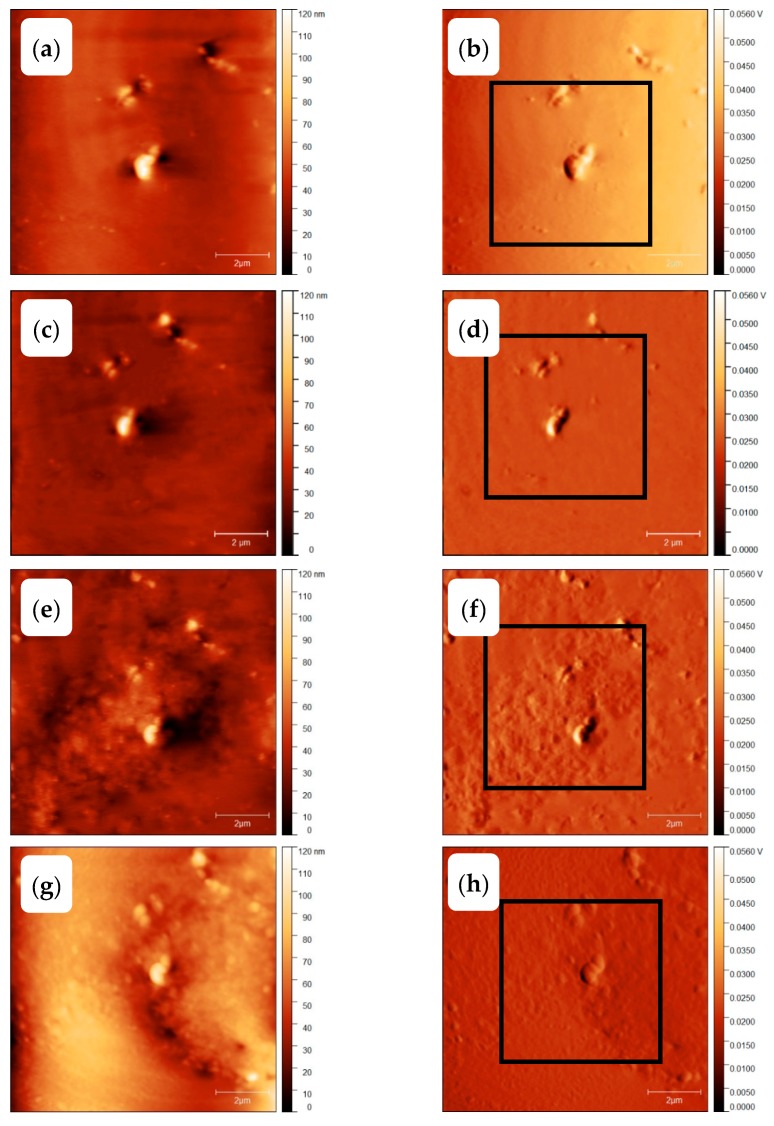
AFM images of PBT with 10 × 10 μm^2^ scan area. The sample was quenched (**a**,**b**), annealed at 290 K for 10,000 s (**c**,**d**), annealed at 490 K for 1 s (**e**,**f**) and annealed at 290 K for 10,000 s and at 490 K for 1 s (**g**,**h**). The left images (**a**,**c**,**e**,**g**) show the topology and the right images (**b**,**d**,**f**,**h**) amplitude differences.

**Figure 16:**
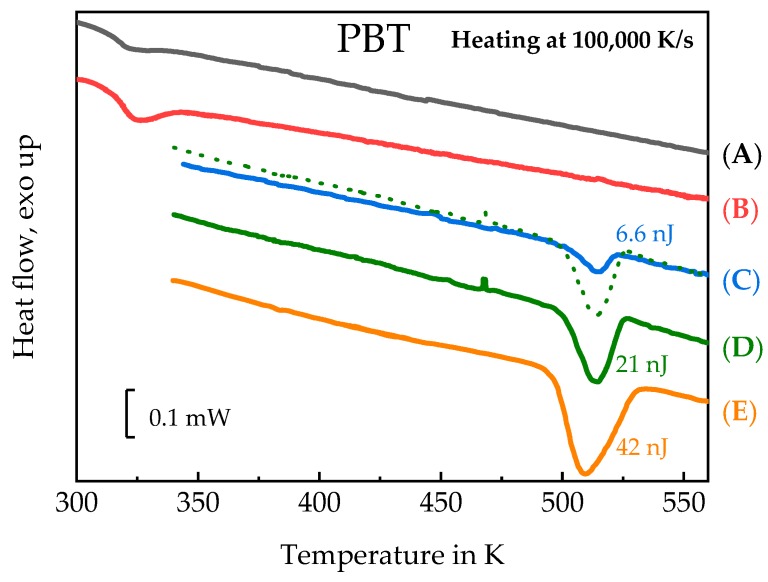
FSC melting curves at heating rate of 100,000 K/s. (**A**)—Quenched sample. (**B**)—Annealing at 290 K for 10,000 s. (**C**)—Annealing at 490 K for 1 s. (**D**)—Annealing at 290 K for 10,000 s followed by a development stage at 490 K for 1 s. (**E**)—Annealing at 490 K for 10,000 s (The dotted curve overlaid on curve (**C**) is the duplication of curve (**D**) for comparison).

At sufficient fast cooling (>50,000 K/s, [69]) no crystals and nuclei are formed on cooling. To verify the amorphous state of the PBT sample after quenching at 100,000 K/s to ambient temperature of 278 K, the scheme of Figure 14b was used. The corresponding AFM images, Figure 15a,b, show some particles, probably dust or other solid impurities, in an otherwise featureless surrounding. These particles allow for an easy judgement of the AFM probe-positioning stability regarding the scan area after repeated heating and cooling scans, (see right column of Figure 15). The heating scan after taking this AFM image is presented as curve (A) in Figure 16. At a heating rate of 100,000 K/s only the glass transition at about 310 K is seen and neither cold crystallization nor melting peaks are detected. In combination with the information provided in Supplementary Materials A3, it was ensured that cooling at 100,000 K/s produced a “nuclei free” amorphous sample.

Next, the morphology of the PBT sample after nucleation at 290 K for 10,000 s without any growth stage, see temperature–time profile shown in Figure 14c, was investigated. The AFM images are shown in Figure 15c,d. Again, only the particles in an unstructured surrounding are seen. The heating scan at 100,000 K/s is shown as curve (B) in Figure 16. The corresponding heating scan at 10,000 K/s after annealing at 290 K for 10,000 s shows the glass transition, cold crystallization and subsequent melting of the just crystallized material (Figure S10, curve (B)), which was not seen without annealing at 290 K, (Figure S10, curve (A)), proving the creation of nuclei during the annealing at 290 K for 10,000 s. However, in Figure 16 curve (B), the heating scan at 100,000 K/s does not show cold crystallization, suggesting that no growth occurs for heating at 100,000 K/s. Application of Tammann’s two-stage nuclei development method therefore seems justified.

As a last test to check the applicability of Tammann’s two stage scheme, the morphology of a melt-crystallized sample, that is, of a sample which was not subject to prior nucleation near *T*_g_, was checked, see temperature program in Figure 14d. Since in bulk polymers of sufficient volume always heterogeneities are present, growth from these heterogeneous nuclei is expected [2]. The AFM images are shown in Figure 15e,f. Besides the dust particles, some micrometer-sized structures appear. The black square in Figure 15f marks the edges of some of them. From the AFM image one gets the impression that, in particular, surface crystals are formed. This is supported by the heating scan after this treatment, curve (C) in Figure 16. Integration of the peak results in a crystallinity of ca. 7%. At a heating rate of 100,000 K/s cold crystallization and recrystallization of isothermally formed crystals is prevented [69]. The melting peak after annealing for 1 s at 490 K without previous nucleation is significantly smaller compared to the melting peak after additional nucleation at 290 K for 10,000 s; curve (D) and the dashed line overlaid on curve (C) in Figure 16. Sample mass was estimated from the measured heat capacity and known specific heat capacity of the fully amorphous sample from ATHAS databank and equals ca. 0.5 ng. After pre-nucleation (curve (D)), crystallinity was ca. 29%. The crystallinity without pre-nucleation (curve (C)) was ca. 9%. The crystallinity after annealing for 10,000 s at 490 K (curve (E)) was ca. 60%.

Finally, we apply Tammann’s two-stage nuclei development scheme to PBT, Figure 14a. The sample is quenched from the melt to the nucleation temperature of 290 K, held there for 10,000 s and then heated to 490 K for 1 s, allowing the formed nuclei to develop to crystals. In Figure 15g,h a much finer crystalline morphology appears, as compared to Figure 15e,f. The number of objects is significantly increased by the preceding nucleation stage. Crystallization now proceeds not only at the sample surface, but seems to occupy the whole volume, as can be judged by comparing curves (D) and (E) in Figure 16. Curve (E) corresponds to the maximum possible crystallinity for annealing at 490 K. After nucleation for 10,000 s at 390 K and development for 1 s at 490 K about 50% of the maximum possible crystallinity is reached.

**Figure 17:**
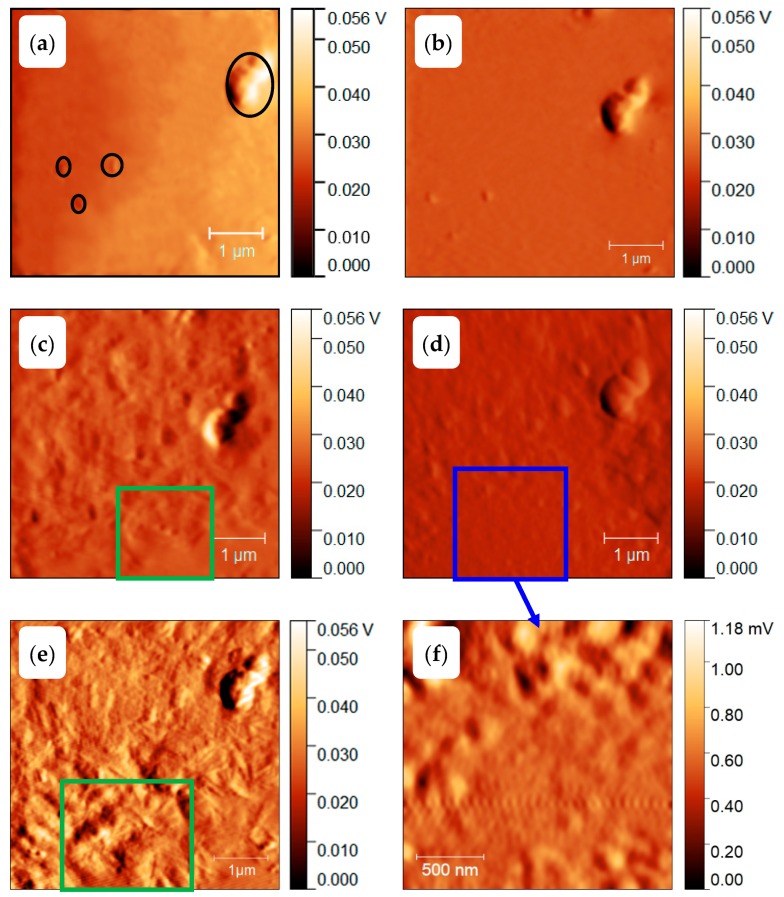
Zoomed-in AFM amplitude images from Figure 15. The images (**a**–**d**) are zoomed-in images of Figure 15b,d,f,h, respectively. The image (**e**) is the morphology that PBT melt-crystallized at 490 K for 2 s. The image (**f**) is a further zoom-in from image (**d**) showing a granular morphology with particle sizes of order 100 nm.

Figure 17 shows sections of 5 × 5 μm^2^ area from the amplitude images of Figure 15. The quenched sample, Figure 17a,b, does not show any structure except the dust particle (marked by black opening) used as a location In Figure 17c spherulites of about 2 µm diameter can be observed. They are marked by the green square. However, with annealing at 290 K for 10,000 s before annealing at 490 K for 1 s, a much finer morphology appears in Figure 17d. At further zooming-in, Figure 17f, the size of such granular structures can be estimated being of the order of 100 nm. The number of crystals can be counted as about 40 in the 2 × 2 μm^2^ area. Details of crystal number determination are presented in the Supplementary Materials. Contrary to the essentially homogeneous nodular morphology with sizes of about 10 nm observed after crystallization at 343 K in reference [8], here the structure is much coarser and much more diverse. In Figure 17f particle sizes are between 20 and 200 nm, indicating a non-uniform growth. Estimating the nuclei density by assuming an average distance between nuclei of 100 nm we end up with about 10^12^ nuclei per mm^3^. This is still a high nuclei density, but about 3 orders of magnitude smaller than reported in reference [8]. In Figure 17e, the PBT was melt-crystallized at 490 for 2 s, which shows spherulites in a clearer way (marked by green square).

## 4. Summary

The combined AFM-FSC device allows for a detailed study of morphology development by AFM after annealing for very different times (from ms to days) and cooling and heating in a very wide range of scanning rates (up to 1,000,000 K/s). The performance of the AFM-FSC device was tested with different polymer samples. Crystallization pathways were widely varied to facilitate, for example, heterogeneous and homogeneous crystal nucleation. Such studies are not limited to crystallizing polymers but any other system, giving some contrast in AFM. The FSC does not only provide a very fast hot stage for the AFM; it also allows studying the thermal behavior of the sample just after taking the AFM image. Furthermore, the micrometer sized hot-spot of the FSC chip and total heating powers of the order of less than 10 mW do not disturb the AFM too much. Thermal treatments can be repeated either by applying the same thermal treatment for improving statistical relevance of the results, or with changing parameters to obtain some dependencies. In both cases, the high reproducibility of the image location is very beneficial.

## Figures and Tables

**Table 1:** Details of samples used for AFM-FSC experiments.

Polymer Sample	Abbreviation	Molar Mass (g/mol)	*M*_w_/*M*_n_	$T_{m}^{0}$ (K)	Grade	Supplier
Poly(butylene terephthalate)	PBT	558,600	2.25	518 [49]		Toray Industry, Inc, Japan
Poly(ether ether ketone)	PEEK	85,000		668 [50]	150 G	Victrex plc. United Kingdom
Polyamide 66	PA 66	18,000		535 [51]	Zytel 101L	DuPont, USA
Poly(ε-caprolactone)	PCL	20,000	1.73	342 [52]		Sigma-Aldrich, USA

*M*_w_: weight-average molar mass; *M*_n_: number-average molar mass; $T_{m}^{0}$: equilibrium melting temperature, from ATHAS databank [49,50,51,52].

## Supplementary Materials

The following are available online at https://www.mdpi.com/2073-4360/11/5/890/s1, Figure S1. AFM images of PA 66 showing the increase of accessible area. Imaging using sample center (green square) to set the reference amplitude—a and b. Imaging using optimized area (yellow square) to set the reference amplitude—c and d. Figure S2. AFM series of images during crystallization of PA 66 at 480 K. Figure S3. Spherulite growth rate estimation in PA 66, isothermally crystallizing at 480 K. Figure S4. AFM series of images during crystallization of PBN at 460 K. Lines are drawn for visualization of spherulite sizes. Figure S5. Spherulite growth rate estimation in PBN, isothermally crystallizing at 460 K. Figure S6. AFM series of images during crystallization of PBT at 450 K. Lines are drawn for visualization of spherulite sizes. Figure S7. Spherulite growth rate estimation in PBT, isothermally crystallizing at 450 K. Figure S8. Temperature profile of pre-check the sample of PBT by annealing at 290 K for in total 10,000 s. Figure S9. Heating of PBT after quenching at 70,000 K/s (A) and after annealing at 290 K for about 10,000 s (B). The integration was done in OriginLab™ using a straight line as the baseline. Figure S10. AFM amplitude (left) and phase (right) images of PBT before and after annealing at 290 K for 10,000 s. Intermediate images are omitted, because there are no changes seen.
