# Supplementary material for: Visualization of Polymer Crystallization by In Situ Combination of Atomic Force Microscopy and Fast Scanning Calorimetry
Source: Polymers (Basel). 2019 May 15;11(5):890. doi: 10.3390/polym11050890 (PMC6572680; doi:10.3390/polym11050890)
Supplement: Supplementary file 1 [file polymers-11-00890-s001.pdf]

Supplementary Materials

Visualization of Polymer Crystallization by in situ Combination of Atomic Force Microscopy and Fast Scanning Calorimetry

Rui Zhang [1], Evgeny Zhuravlev [1, 2, 3]*, René Androsch [4] and Christoph Schick [1, 5]

[1] Institute of Physics and Competence Centre CALOR, University of Rostock, 18051 Rostock, Germany; rui.zhang@uni-rostock.de
[2] Department of Polymer Science and Engineering, School of Chemistry and Chemical Engineering, Key Laboratory of High-Performance Polymer Materials and Technology of Ministry of Education, and The State Key Laboratory of Coordination Chemistry, Nanjing University, Nanjing 210093, P. R. China; evgeny.zhuravlev@uni-rostock.de
[3] Sheyang Research Institute, Nanjing University, Sheyang, 224300, P. R. China; evgeny.zhuravlev@uni-rostock.de
[4] Interdisciplinary Center for Transfer-oriented Research in Natural Sciences (IWE TFN), Martin Luther University Halle-Wittenberg, 06099 Halle/Saale; rene.androsch@iw.uni-halle.de
[5] Butlerov Institute of Chemistry, Kazan Federal University, 18 Kremlyovskaya street, Kazan 420008, Russian Federation; christoph.schick@uni-rostock.de
* Correspondence: evgeny.zhuravlev@uni-rostock.de;

Supplementary materials includes images, that authors decided not to include in the main text, but may be useful for readers, interested in particular details.

A.1 Increase of accessible area for AFM

Improved Z-range of the used AFM was reached by optimizing reference amplitude setting area. The result of optimization can be seen from Figure S1.

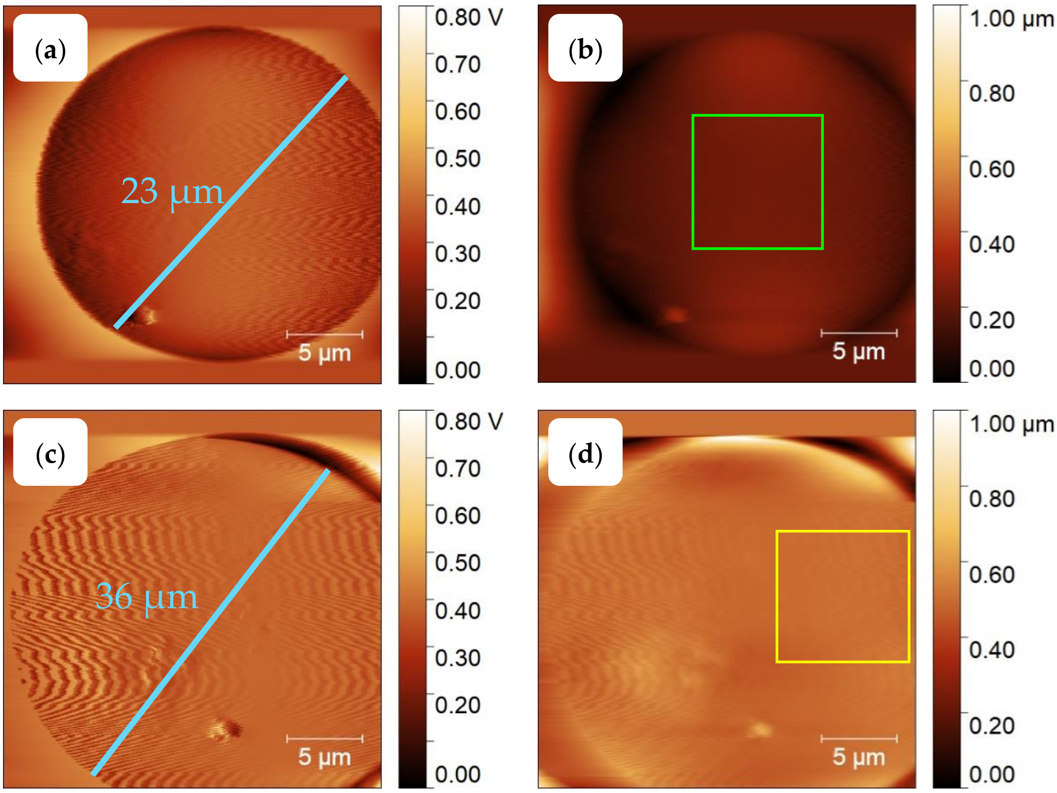

Figure S1. AFM images of PA 66 showing the increase of accessible area. Imaging using sample center (green square) to set the reference amplitude – **a** and **b**. Imaging using optimized area (yellow square) to set the reference amplitude – **c** and **d**.

A.3 Growth rates of PA66, PBN and PBT

Additional figures for determination of spherulite growth rate for various polymers are collected in this part. Figure S2 and Figure S3 show crystallization of PA 66. Figure S4 and Figure S5 – crystallization of PBN. And Figure S6 and Figure S7 – crystallization of PBT.

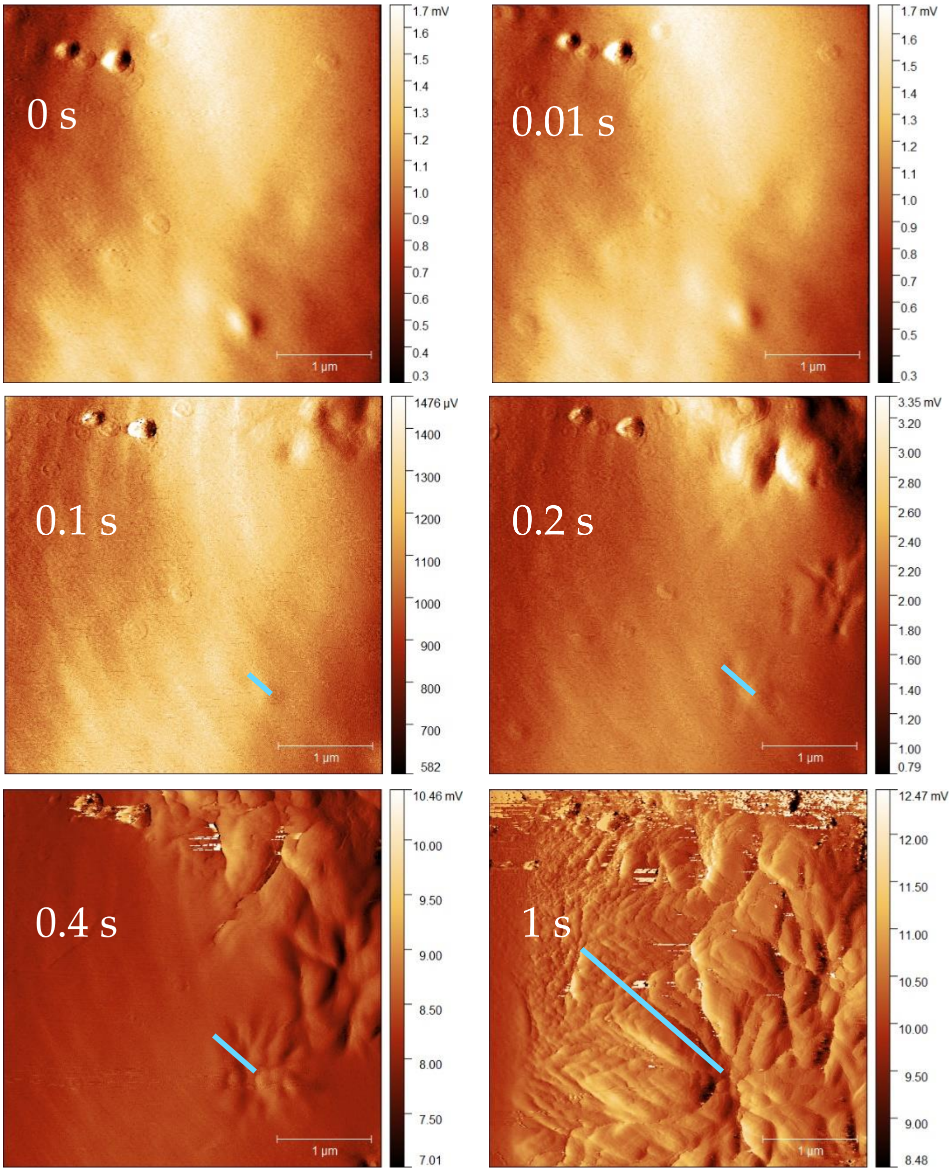

Figure S2. AFM series of images during crystallization of PA 66 at 480 K.

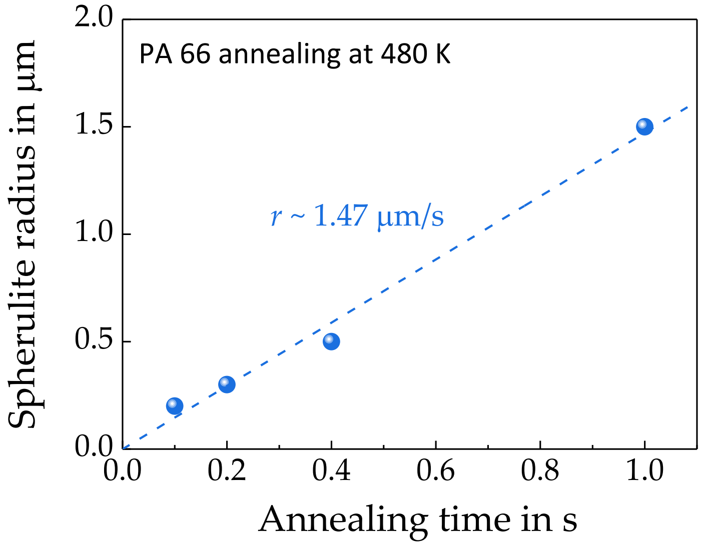

Figure S3. Spherulite growth rate estimation in PA 66, isothermally crystallizing at 480 K.

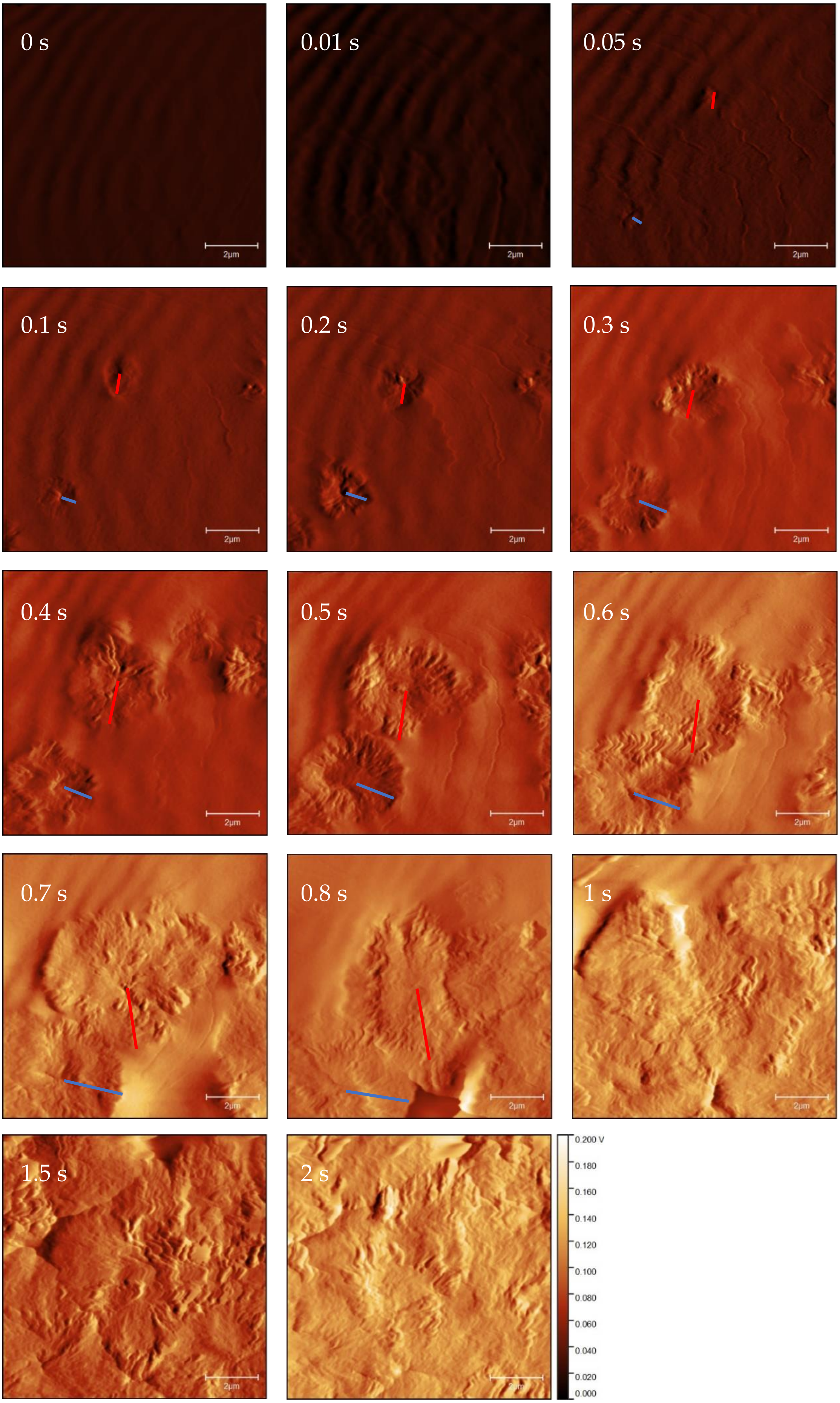
0 s
0.01 s
0.05 s
0.1 s
0.2 s
0.3 s
0.4 s
0.5 s
0.6 s
0.7 s
0.8 s
1 s
1.5 s
2 s
2μm
0.200 V
0.180
0.160
0.140
0.120
0.100
0.080
0.060
0.040
0.020
0.000

Figure S4. AFM series of images during crystallization of PBN at 460 K. Lines are drawn for visualization of spherulite sizes.

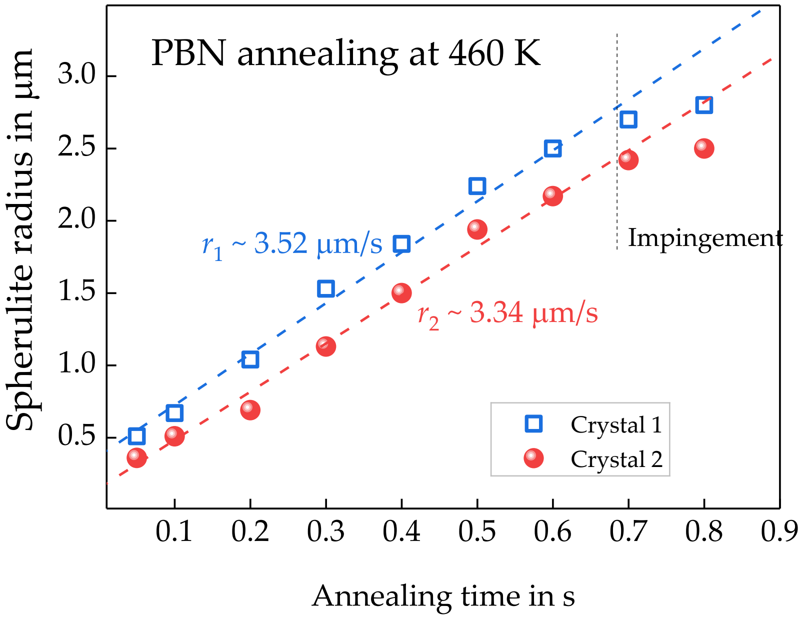

Figure S5. Spherulite growth rate estimation in PBN, isothermally crystallizing at 460 K.

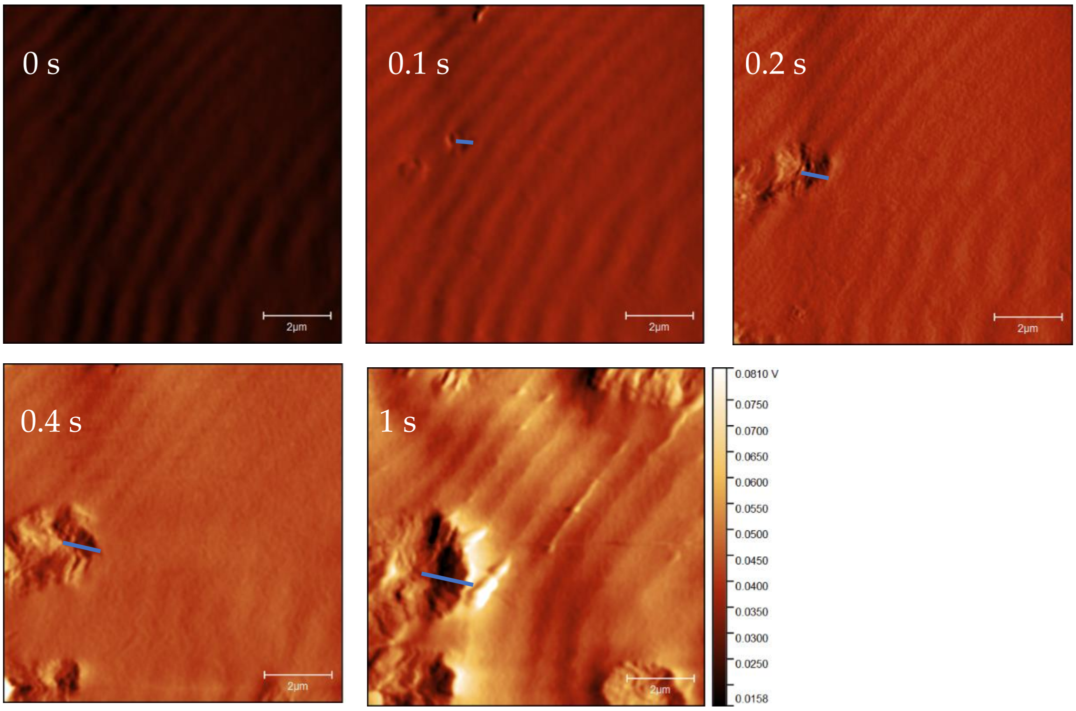

Figure S6. AFM series of images during crystallization of PBT at 450 K. Lines are drawn for visualization of spherulite sizes.

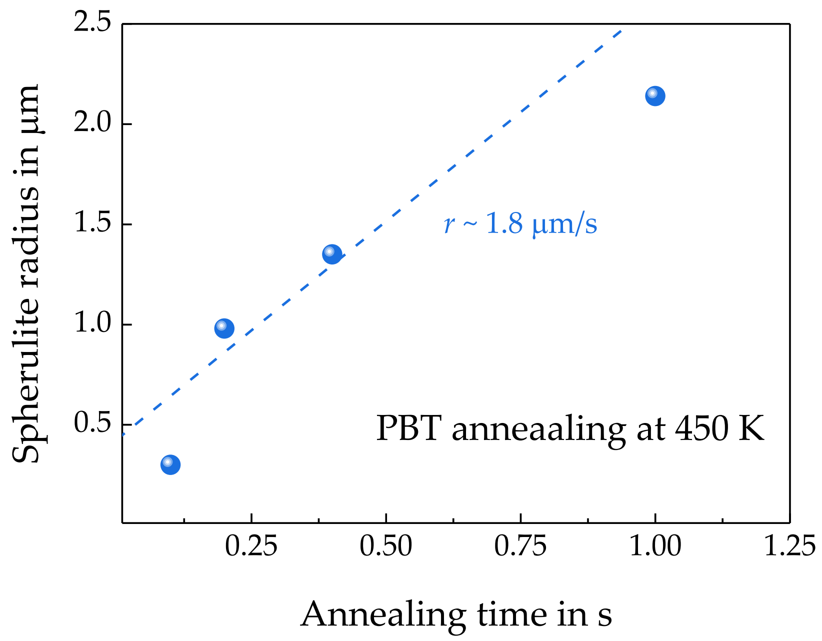

Figure S7. Spherulite growth rate estimation in PBT, isothermally crystallizing at 450 K.

A.5 Annealing of PBT at 290K for 10,000 s

Prior to Tammann's experiment on PBT, summarized in Figure 14 in the main text, the sample was checked for absence of crystallization at 290 K. For that, the AFM images were captured after 1,000 s, 4,000 s and 5,000 s annealing. The scheme of the experiment is shown in Figure **S8**.

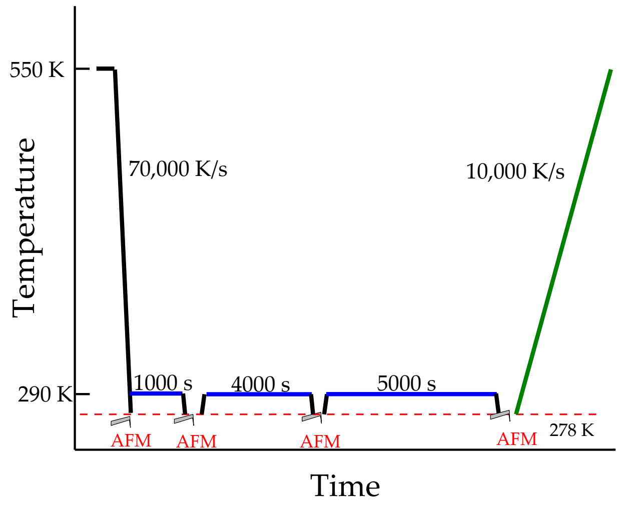

Figure S8. Temperature profile of pre-check the sample of PBT by annealing at 290 K for in total 10,000 s.

Figure S9 shows the heating curves of as quenched (A) and PBT after annealing at 290 K for 10,000 s (B). Curve (A) shows a glass transition step and no cold crystallization and melting peaks, indicating the "nuclei free" amorphous sample. After 10,000 s, there were one crystallization and a following melting peak after the glass transition. The crystallization and melting peaks have been integrated and their total area was nearly zero, suggesting no previous crystallization.

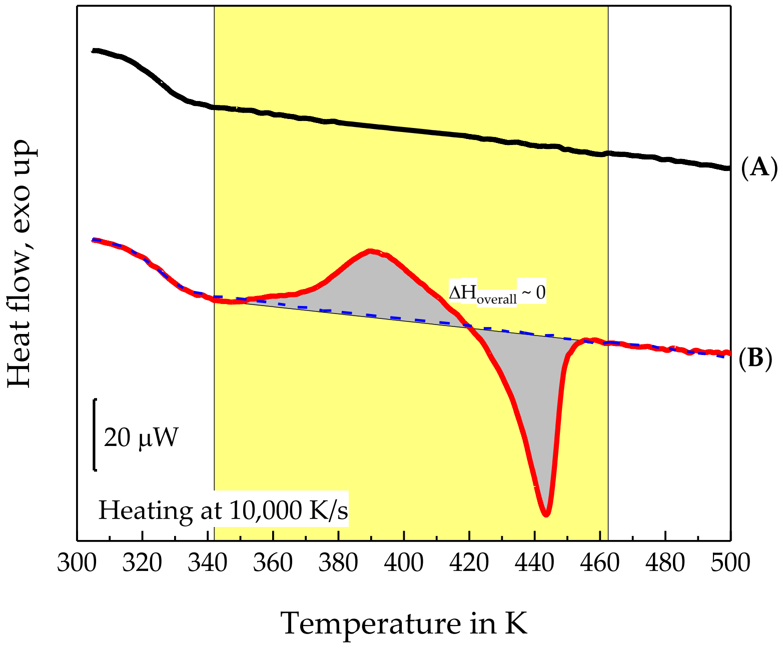

Figure S9. Heating of PBT after quenching at 70,000 K/s (**A**) and after annealing at 290 K for about 10,000 s (**B**). The integration was done in OriginLab™ using a straight line as the baseline.

Figure S10 shows AFM images of as-quenched and PBT after annealing at 290 K for different times. All images did not show any structures and these results lead to the conclusion in section 4.3 in the main text that there was no crystallization at 290 K within 10,000 s. Combining with the data in Figure S9, it can be proposed that there was only a nucleation process during annealing at 290 K for times below 10,000 s, but no crystal growth.

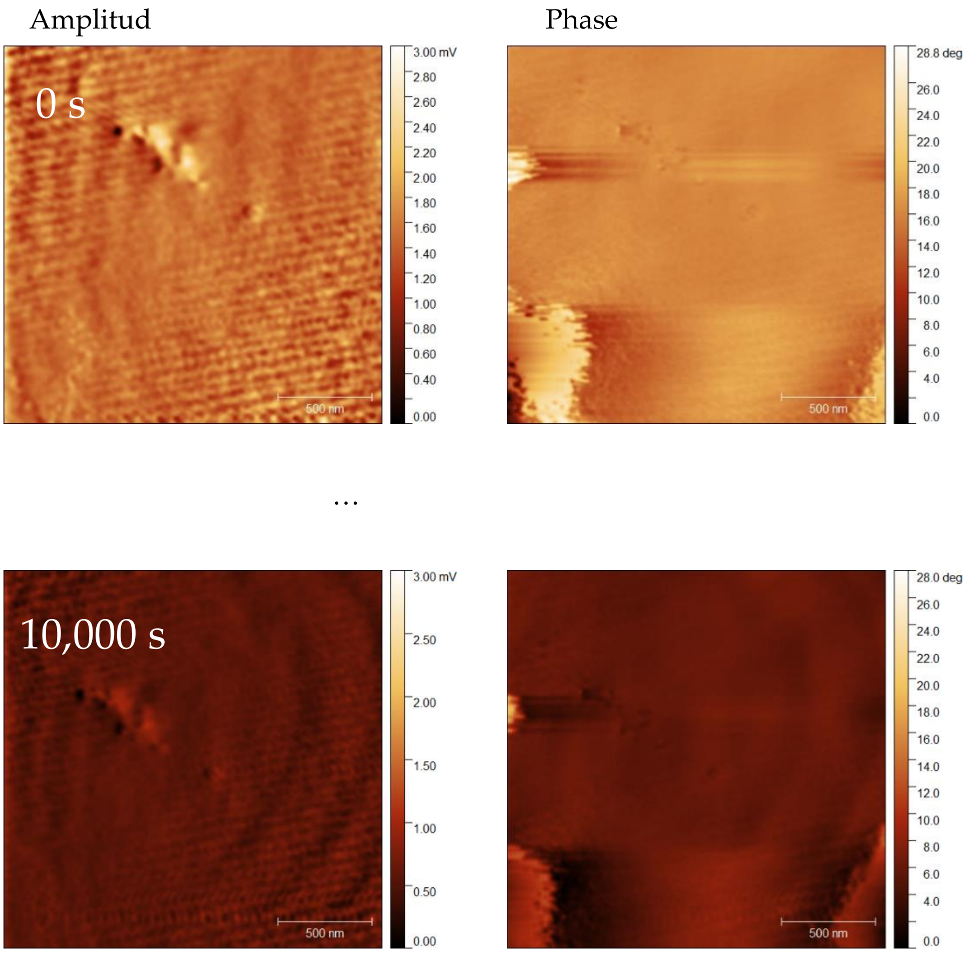

Figure S10. AFM amplitude (left) and phase (right) images of PBT before and after annealing at 290 K for 10,000 s. Intermediate images are omitted, because there are no changes seen.
